# Pharmacokinetics of HLD200, a Delayed-Release and Extended-Release Methylphenidate: Evaluation of Dose Proportionality, Food Effect, Multiple-Dose Modeling, and Comparative Bioavailability with Immediate-Release Methylphenidate in Healthy Adults

**DOI:** 10.1089/cap.2018.0122

**Published:** 2019-04-03

**Authors:** Tao Liu, Jogarao V.S. Gobburu, Michelle D. Po, Angus McLean, Norberto J. DeSousa, Floyd R. Sallee, Bev Incledon

**Affiliations:** ^1^ Center for Translational Medicine, School of Pharmacy, University of Maryland, Baltimore, Maryland.; ^2^ Highland Therapeutics Inc., Toronto, Ontario, Canada.; ^3^ Ironshore Pharmaceuticals and Development, Inc., Camana Bay, Cayman Islands.

**Keywords:** methylphenidate, attention-deficit/hyperactivity disorder, pharmacokinetics, relative bioavailability, food effect, dose proportionality

## Abstract

***Objectives:*** HLD200, an oral, once-daily, evening-dosed, delayed-release, and extended-release methylphenidate (DR/ER-MPH), was designed to provide efficacy from the early morning, throughout the day, and into the evening to individuals with attention-deficit/hyperactivity disorder. The objectives were to evaluate DR/ER-MPH pharmacokinetic (PK) properties in healthy adults, including dose proportionality, food effect, the potential of accumulation using multiple-dose modeling, and bioavailability compared to an immediate-release MPH (IR MPH).

***Methods:*** Three open-label, single-dose, crossover studies were conducted, all with a 7-day washout between treatments. In Study I, 20 subjects received evening-dosed DR/ER-MPH (20 and 100 mg) followed by a medium-fat breakfast; 13 subjects received a subsequent 100-mg dose of DR/ER-MPH followed by a low-fat breakfast. In Study II, 18 subjects were evaluated after receiving evening-dosed DR/ER-MPH (100 mg) under 3 conditions: immediately after a high-fat meal, sprinkled on applesauce, and in a fasted state. In Study III, 11 and 12 subjects received evening-dosed DR/ER-MPH (100 mg) and morning-dosed IR MPH (20 mg), respectively.

***Results:*** DR/ER-MPH demonstrated dose proportionality between 20- and 100-mg doses. DR/ER-MPH PK parameters were not significantly affected by breakfast content or by sprinkling capsule contents. A high-fat meal immediately preceding evening dosing did not affect total MPH exposure but lowered peak MPH exposure by 14% and 11% versus fasted and sprinkled states, and time to peak exposure was delayed by ∼2.5 hours; these PK differences are unlikely to be clinically significant. Based on multiple-dose simulations using data from Study I, negligible accumulation of DR/ER-MPH was predicted. The relative bioavailability for DR/ER-MPH compared to IR MPH was 73.9%. No serious adverse events (AEs) were reported, and the observed AEs were consistent with MPH. There were no discontinuations in Studies I and III, but three participants withdrew in Study II due to AEs.

***Conclusions:*** Evening-dosed DR/ER-MPH demonstrated dose proportionality and can be administered with or without food. Significant accumulation is unlikely with multiple dosing.

## Introduction

Long-acting stimulants are recommended as first-line pharmacotherapy for attention-deficit/hyperactivity disorder (ADHD), with methylphenidate (MPH) frequently prescribed to children and adolescents with ADHD (Pliszka et al. 2007; Wolraich et al. 2011). Existing extended-release (ER) formulations of MPH are administered once daily in the morning and release varying proportions of MPH throughout the day. The availability of products with unique pharmacokinetic (PK) profiles, differing in the onset and duration of action, peak plasma levels, and rates of release, allows physicians greater ability to tailor therapy to the symptom profile of their patients (Maldonado 2013; Childress 2016). Despite this, clinically meaningful control of ADHD-associated early morning functional impairment and symptoms from the time of awakening until arrival at school, but not at the expense of efficacy later in the day, remains a significant unmet need in stimulant-treated youth with ADHD (Sallee 2015; Faraone et al. 2017). Some MPH formulations have a delay in the initial onset of action of up to 2 hours (Childress 2016), which can result in inadequate symptom control and impaired functioning during the before-school early morning routine, a particularly challenging time of day for school-aged children with ADHD and their families (Whalen et al. 2006; Sallee 2015; Faraone et al. 2017).

HLD200, a delayed-release and ER MPH (DR/ER-MPH) formulation, was specifically designed to provide ADHD control that starts upon awakening and lasts into the evening. DR/ER-MPH capsules contain uniform microbeads composed of two functional film coatings, a DR and an ER layer, surrounding an immediate-release (IR) MPH core. DR/ER-MPH is dosed in the evening, and provides a consistent and predictable 8- to 10-hour delay in the initial release of MPH followed by an extended monophasic pattern of release throughout the day (Childress et al. 2018). Early morning (before school) to evening efficacy was demonstrated in a pivotal phase 3 trial in children aged 6–12 years with ADHD, in which 3 weeks of DR/ER-MPH treatment resulted in significant improvements not only in ADHD symptoms but also in early morning and late afternoon/evening functional impairment, with treatment being generally well tolerated (Pliszka et al. 2017).

Clinical pharmacology is evaluated early in product development to inform dosing recommendations in later phase trials and product labeling. The PK properties of the to-be-marketed DR/ER-MPH formulation were evaluated in three single-dose studies in healthy adult volunteers. The objectives were to assess the following: (1) dose proportionality between the lowest and highest commercially proposed doses of DR/ER-MPH (Study I); (2) the effect of morning and evening food content as well as sprinkling capsule contents on the PK profile of DR/ER-MPH (Studies I and II); (3) the potential of drug accumulation using multiple-dose PK modeling (data from Study I); and (4) the bioavailability of DR/ER-MPH compared to IR MPH (Study III).

## Methods

### Study conduct

Three independent phase 1, single-dose, open-label crossover studies were conducted in healthy adults. All studies were conducted at a single clinical center (Prism Clinical Research, LLC., St. Paul, MN) in accordance with the Declaration of Helsinki, Good Clinical Practice guidelines of the International Conference on Harmonization, and all applicable local/country-specific laws and regulations. Study protocols and informed consent forms were reviewed and approved by an Institutional Review Board for the investigational site in accordance with the United States (U.S.) Food and Drug Administration (FDA) regulations set forth in the Code of Federal Regulations Title 21, Part 56. Informed consent was collected from all participants before enrollment.

### Participants

Eligible participants were healthy males and females aged 18–55 years with a body mass index of 18.5–30 kg/m^2^ in Study I and 18–32 kg/m^2^ in Studies II and III. All other inclusion and exclusion criteria were consistent across the three studies. Inclusion criteria included but were not limited to the following: (1) general good health with no significant findings by physical examination, laboratory values, or electrocardiogram (ECG); and (2) female participants were required to have a negative urine pregnancy result, and those of childbearing potential were required to practice effective contraception during the study and be willing to continue contraception for 90 days after their last dose of study treatment.

Exclusion criteria included but were not limited to the following: (1) history or presence of clinically significant cardiovascular, pulmonary, hepatic, renal, hematologic, gastrointestinal (including narrowing of the gastrointestinal tract), endocrine, immunologic, dermatologic, neurologic, oncologic, or psychiatric disease or any other condition that, in the opinion of the principal investigator, would jeopardize the safety of the participant or the validity of the study results; (2) history of glaucoma; (3) history of psychiatric or neurologic conditions of clinical significance, such as mood disorders, depression, schizophrenia, and other psychotic disorders, anxiety, ADHD, seizures (except febrile seizures as a child), motor tics, or a current diagnosis or family history of Tourette's syndrome; (4) recent history or current evidence of illicit or prescription drug abuse or alcohol abuse; (5) history of any condition that may interfere with the absorption, distribution, metabolism, or excretion of MPH; (6) positive history of HIV, hepatitis B, or hepatitis C; and (7) participation in a clinical trial with an investigational drug within 30 days preceding study enrollment.

Participants were required to abstain from dietary supplements, vitamins, herbal medications, antacids, prescription drugs (other than contraceptives), nonprescription drugs taken for nontherapeutic indications, broccoli, brussels sprout, grapefruit, and Seville oranges for 7 days before clinical research unit (CRU) admission through the end of the study. For 3 days before each CRU admission to the end of the study, participants agreed not to consume alcohol and xanthine-, caffeine-, or poppy-containing products. In addition, participants agreed to refrain from strenuous physical activity outside of their normal daily routine for 2 days before CRU admission.

### Study design

All three studies examined the to-be-marketed formulation of DR/ER-MPH in healthy adults. The to-be-marketed formulation of DR/ER-MPH, which was used in the pivotal phase 3 DR/ER-MPH studies (Pliszka et al. 2017 and NCT02493777), differs only in the manufacturing process used to create the MPH core compared to the MPH00400 formulation described in the previous PK study (Childress et al. 2018). The dissolution profiles of the to-be-marketed and MPH00400 formulations were similar based on the similarity factor (f2) analysis (data on file), meeting the FDA guidance criteria (US Food and Drug Administration 1997). For all studies, participants were admitted to the CRU the day before dosing, and remained in the CRU for another 48 and 24 hours after DR/ER-MPH and IR MPH dosing, respectively, for PK sampling and safety assessments. Treatments were provided with 240 mL of room temperature water, and water was allowed as needed except for 1 hour after drug administration. The washout period between each treatment period was 7 days (± 1). Participants in Studies I and II returned to the CRU for final safety assessments on day 13 (± 1) for Study I Part 1, day 6 (± 1) for Study I Part 2, and day 19 (± 1) for Study II.

Study I, conducted in two parts from June to July 2015, evaluated dose proportionality and the effect of breakfast on MPH PK parameters after evening-dosed DR/ER-MPH. Part 1 used a Latin Square, two-sequence, two-period, crossover study design, in which DR/ER-MPH was administered at doses of 100 or 20 mg at ∼8:00 PM in a fasted state of at least 8 hours; participants received a medium-fat (low- to moderate-fat [20.9–31.3% of calories from fat]/high-calorie [690 kcal]) breakfast ∼12 hours later. In Part 2, 13 participants from Part 1 were subsequently administered 100 mg of DR/ER-MPH in a fasted state followed by a low-fat (low-fat [16.4% fat]/low-calorie [178.5 kcal]) breakfast ∼12 hours later. A nonfat, 128 kcal evening snack was provided ∼2 hours after each dose.

Study II, conducted from May to July 2015, evaluated the PK profile of DR/ER-MPH in relation to food intake at evening administration. Using a Latin Square, six-sequence, three-way crossover study design, participants were randomized to receive a single 100-mg dose of DR/ER-MPH at 9:00 PM under the following three conditions: fed (intact capsule administered after a high-fat test meal in accordance with the FDA guidelines of ∼50% fat and 800–1000 kcal [US Food and Drug Administration 2002], beginning 30 minutes and ending 5 minutes predose); fasted (intact capsule administered after a ≥8 hour fast); and sprinkled (capsule contents sprinkled on applesauce after a ≥8 hour fast). No evening snack was given, and all participants consumed a standard high-fat (∼50%)/high-calorie (800–1000 kcal) breakfast at ∼8:00 AM the following morning after dosing.

Study III, conducted in July 2016, evaluated the relative bioavailability of evening-administered DR/ER-MPH versus morning-administered IR MPH (Ritalin^®^), with both formulations administered in the fasted state. Subjects were randomized to receive the following treatments in a crossover manner: (1) a single 100-mg capsule of DR/ER-MPH administered at ∼9:00 PM, 3 hours after a low-fat meal, with a standard breakfast 12 hours postdose (no evening snack) and standard meals resuming 16 hours postdose; (2) a single 20-mg tablet of IR MPH administered ∼8:00 AM after an overnight (∼10 hour) fast, with standard meals resuming 4 hours postdose.

### Sample preparation and analytical methods

After DR/ER-MPH administration, blood samples (4 mL) for PK analyses were collected at the following time points in all three studies: predose (5 minutes before dosing) and 2, 4, 6, 8, 8.5, 9, 9.5, 10, 10.5, 11, 11.5, 12, 13, 14, 15, 16, 17, 18, 19, 20, 22, 24, 36, and 48 hours postdose (±2 minutes). For Study III, blood samples after IR MPH administration were collected at the following time points: predose and 0.25, 0.5, 1, 1.5, 2, 2.5, 3, 3.5, 4, 5, 6, 8, 10, 12, 14, 17, 20, and 24 hours postdose (±2 minutes). Blood samples were collected into prechilled sodium fluoride/potassium oxalate vacutainers, placed on ice, and within 30 minutes from collection, centrifuged at 3000 rpm at 4°C for 10 minutes. The resulting plasma samples were transferred into two prechilled tubes and stored at −70°C before shipping for analysis.

MPH concentrations for plasma samples were analyzed by validated, high-performance liquid chromatography tandem mass spectrometry (BioPharma Services, Toronto, Canada). Calibration curves were determined by least-squares (LS) linear regression analysis (weighted 1/ × ^2^) on MPH-d_9_ calibration standards. Calibration curves were linear in the range of 0.02–20 ng/mL. Mean correlation coefficients for the regressions were 0.9992, 0.9991, and 0.9988 for Studies I, II, and III, respectively. Interassay precision, as measured by the coefficient of variation (CV) for calibration standards, ranged from 0.0% to 2.1%, 0.0% to 2.4%, and 0.0% to 3.1% for Studies I, II, and III, respectively. For the quality control samples (0.06, 1.6, 10, and 16 ng/mL), interassay precision ranged from 0.0% to 4.5% and 0.0% to 2.1%, and 3.2% to 8.1% for Studies I, II, and III, respectively. In all studies, plasma samples below the limit of quantitation (0.02 ng/mL) were assigned values of zero.

### PK analysis

In Studies I and II, the primary study endpoint was the PK profile (i.e., the rate and extent of MPH absorption) of a single dose of DR/ER-MPH. PK parameters, calculated using standard noncompartmental analysis, included the plasma concentration area under the curve (AUC) from time zero (predose) to the last quantifiable drug concentration (AUC_0-t_), AUC from zero extrapolated to infinity (AUC_0-∞_), maximum plasma drug concentration (C_max_), time to reach maximum concentration (T_max_), terminal elimination rate constant (λ_z_), and terminal half-life (t_1/2_). AUC_0-t_ was calculated by the linear trapezoidal rule. AUC_0-∞_ was calculated from AUC_0-t_ + C_t_/λ_z_, where C_t_ is the last quantifiable concentration. In Study III, the primary study endpoint was the relative bioavailability of DR/ER-MPH to IR MPH based on dose-normalized (DN) AUC_0-t_.

### Statistical analysis

Noncompartmental analyses of PK data and statistical analyses of bioequivalence and dose proportionality were performed using Phoenix WinNonlin v6.3 or v6.4 (Certara, Princeton, NJ). Descriptive statistics were calculated for quantitative parameters using SAS^®^ v9.2 or higher (SAS Institute, Inc., Cary, NC). For bioequivalence evaluations, a mixed-effects model analysis based on the U.S. FDA Guidance for Industry (2001) “*Statistical Approaches to Establishing Bioequivalence”* was performed on the natural logarithmic (Ln) transformation of the primary PK exposure metrics C_max_ and AUC_0-t_. The mixed-effects model included sequence, period, and treatment as fixed effects and subject as a random effect. For the dose proportionality analysis, DN C_max_ and AUC_0-t_ values from Study I (Part 1) were used for the bioequivalence evaluation, as described above. Per FDA guidance, exposure equivalence was concluded if the 90% confidence intervals (CIs) for the test/reference ratio of geometric LS means for the Ln-transformed C_max_ and AUC_0-t_ fell within the bioequivalence limits of 0.8–1.25 (US Food and Drug Administration 2001).

All participants who received at least one dose of study drug were included in the safety population. The PK population was defined as all randomized participants who received study drug and for whom the PK profile could be adequately characterized.

### Multiple-dose PK simulation

A one-compartment model with first-order absorption and a lag time was used to describe the mean PK profile of orally administered DR/ER-MPH using the mean concentration–time profile data from Study I (Parts 1 and 2). Mean PK profiles corresponding to multiple once-daily doses of 20 and 100 mg of DR/ER-MPH were simulated to predict the concentration–time profile of DR/ER-MPH at steady state and assess accumulation. The modeling and simulation of DR/ER-MPH PK profiles were conducted in Phoenix NLME 1.3 (Certara, Princeton, NJ). Statistical analyses were conducted using R 3.2.3. Accumulation ratios for C_max_ and C_min_ were calculated by dividing the simulated PK parameter at steady state by the simulated parameter after a single dose.

### Safety

In all three studies, the same general assessments were performed. Safety and tolerability were assessed throughout the studies by spontaneous reporting or observed adverse events (AEs), physical examination, clinical laboratory results, vital signs, ECG, and Columbia Suicide Severity Rating Scale (only administered at screening in Study III). AEs were coded using the Medical Dictionary for Regulatory Affairs, version 17.1 (Studies I/II) or version 18.1 (Study III).

## Results

### Subject disposition and baseline characteristics

Study I enrolled 20 healthy adult volunteers. All 20 (10 in each treatment sequence) completed Part 1 (dose proportionality), and 13 participants were subsequently enrolled in and completed Part 2 (morning food effect). Study II (evening food effect) enrolled 24 healthy adults, and 18 completed the study (3 in each treatment sequence). Of the 6 participants who did not complete Study II, 3 withdrew because of safety reasons and 3 withdrew consent for personal reasons. Study III (comparative bioavailability) enrolled 12 healthy adults (6 in each treatment sequence). All but one participant completed Study III per protocol; this participant withdrew consent for nonsafety reasons before DR/ER-MPH dosing but completed PK assessments after IR MPH dosing. Due to this withdrawal, 11 and 12 subjects were included in the PK populations after a single dose of DR/ER-MPH and IR MPH, respectively. Demographics and baseline characteristics for all studies are presented in Table 1.

**Table 1.** Baseline Demographics and Characteristics

	*Study I part 1 dose proportionality* n* = 20*	*Study I part 2 morning food effect* n* = 13*	*Study II evening food effect* n* = 24*	*Study III relative bioavailability* n* = 12*
Age (years)				
Mean (SD)	26.6 (6.0)	26.5 (6.6)	40.3 (11.0)	28.1 (6.7)
Median (range)	25.5 (18–39)	23.0 (18–39)	44.0 (21–54)	27.5 (20–41)
Gender, *n* (%)				
Male	6 (30.0)	6 (46.2)	15 (62.5)	4 (33.3)
Race, *n* (%)				
White	16 (80.0)	10 (76.9)	15 (62.5)	11 (91.7)
Black	2 (10.0)	2 (15.4)	9 (37.5)	1 (8.3)
Other	2 (10.0)	1 (7.7)	0 (0)	0 (0)
Ethnicity, *n* (%)				
Not Hispanic/Latino	20 (100)	13 (100)	23 (95.8)	12 (100)
Weight at screening (kg)				
Mean (SD)	67.6 (10.9)	69.0 (11.7)	81.5 (13.3)	76.2 (14.7)
Median (range)	65.7 (51.8–90.1)	64.8 (51.8–90.1)	81.4 (57.0–103.7)	74.3 (51.7–95.2)
Body mass index (kg/m^2^)				
Mean (SD)	23.2 (2.1)	23.1 (2.0)	26.8 (3.5)	24.9 (4.0)
Median (range)	23.6 (18.5–26.5)	23.1 (19.3–25.9)	28.1 (19.9–31.7)	25.1 (18.5–31.7)

SD, standard deviation.

### Dose proportionality

**FIG. 1.**
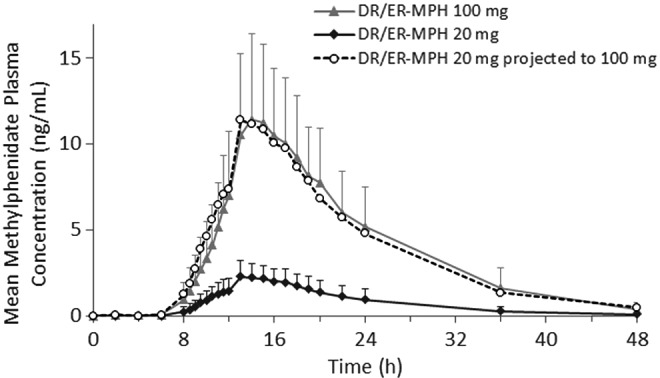
Mean methylphenidate plasma concentrations after single evening doses of 20- and 100-mg DR/ER-MPH followed by a medium-fat breakfast (*n* = 20) in Study I (Part 1). Error bars represent + standard deviation of the mean. DR/ER-MPH, delayed-release and extended-release methylphenidate.

Mean plasma concentration–time profiles after single evening doses of 20- and 100-mg DR/ER-MPH showed that MPH concentration increased in a dose-dependent manner. When the mean 20-mg concentration–time profile was dose normalized to 100 mg, the projected concentration–time profile was highly consistent with the observed mean 100-mg concentration–time profile (Fig. 1). PK parameters after the two doses are presented in Table 2. Early drug exposure from 0 to 10 hours after evening administration (8:00 PM–6:00 AM) was on average 4.2% and 3.2% of total drug exposure for the 20 and 100 mg doses, respectively, indicating minimal MPH absorption during the overnight period spanning the first 10 hours after DR/ER-MPH administration. When the DN 20-mg dose was compared to the 100-mg dose, the geometric LS mean ratios for AUC_0-t_ and C_max_ were 0.964 (90% CI: 0.900–1.031) and 1.042 (90% CI: 0.934–1.162), respectively. These geometric LS mean ratios fell within the standard 0.8–1.25 limits of bioequivalence (Table 3), indicating dose proportionality between the 20- and 100-mg doses of DR/ER-MPH.

**Table 2.** Methylphenidate PK Parameters After Single Evening Doses of 20-mg and 100-mg DR/ER-MPH (Study I, Part 1)

*Parameter*	*20 mg* n* = 20*	*100 mg* n* = 20*
Mean C_max_ (ng/mL) ± CV (%)	2.56 ± 34.4	12.31 ± 36.5
Mean DN C_max_ ([ng/mL]/mg) ± CV (%)	0.1282 ± 34.4	0.1231 ± 36.5
Mean AUC_0-t_ (ng·h/mL) ± CV (%)	33.4 ± 38.9	171.4 ± 33.0
Mean DN AUC_0-t_ ([ng·h/mL]/mg) ± CV (%)	1.67 ± 38.9	1.714 ± 33.0
Mean AUC_0-∞_ (ng·h/mL) ± CV (%)	34.7 ± 40.5	176.7 ± 34.0
Mean T_max_ (h) ± CV (%)	14.35 ± 12.7	14.85 ± 12.0
Median T_max_ (h) (range)	14.00 (13.00–19.00)	14.00 (13.00–20.00)
Mean t_1/2_ (h) ± CV (%)	6.51 ± 32.3	6.40 ± 34.5
Mean λ_z_ (1/h) ± CV (%)	0.1166 ± 29.4	0.1192 ± 29.3

DR/ER-MPH, delayed-release and extended-release methylphenidate; DN, dose-normalized; C_max_, peak observed plasma concentration; AUC_0-t_, area under the concentration–time curve from zero (predose) to time of last quantifiable concentration; AUC_0-∞_, area under the concentration–time curve from zero (predose) extrapolated to infinite time; T_max_, time to peak observed plasma concentration; t_1/2_, terminal phase half-life; λ_z_, terminal phase rate constant; CV, coefficient of variation; PK, pharmacokinetic.

**Table 3.** Bioequivalence Analysis—Studies I, II, and III

		*Geometric LS mean*					
	*Parameter*	*Treatment 1*	*Treatment 2*	*Treatment 3*	*Intrasubject CV%*	*Test / reference*	*Geometric LS mean ratio (90% CI)*
Study I Part 1: dose proportionality		20 mg (*n* = 20)	100 mg (*n* = 20)	—			
	DN C_max_ ([ng/mL]/mg)	0.120	0.116	—	20.06	20 / 100 mg	1.042 (0.934–1.162)^a^
	DN AUC_0-t_ ([ng·h/mL]/mg)	1.57	1.63	—	12.42	20 / 100 mg	0.964 (0.900–1.031)^a^
Study I Part 2: morning food effect		Low-fat breakfast 100 mg (*n* = 13)	Medium-fat breakfast 100 mg (*n* = 13)	—			
	C_max_ (ng/mL)	12.45	12.83	—	NR	Medium-fat breakfast / low-fat breakfast	0.970 (0.773–1.217)
	AUC_0-t_ (ng·h/mL)	161.4	174.8	—	NR	Medium-fat breakfast / low-fat breakfast	0.923 (0.733–1.164)
Study II : evening meal effect		Fed 100 mg (*n* = 18)	Sprinkled 100 mg (*n* = 18)	Fasted 100 mg (*n* = 18)			
	C_max_ (ng/mL)	11.25	12.71	12.99	21.24	Fed / fasted	0.866 (0.769–0.975)
						Sprinkled / fasted	0.978 (0.869–1.102)^a^
						Fed / sprinkled	0.885 (0.786–0.996)
	AUC_0-t_ (ng·h/mL)	161.5	170.7	166.2	16.72	Fed / fasted	0.972 (0.885–1.067)^a^
						Sprinkled / fasted	1.027 (0.936–1.128)^a^
						Fed / sprinkled	0.946 (0.861–1.039)^a^
Study III : comparative bioavailability		DR/ER-MPH 100 mg (*n* = 11)	IR MPH 20 mg (*n* = 12)	—			
	DN C_max_ ([ng/mL]/mg)	0.093	0.329	—	33.6	DR / ER-MPH / IR MPH	0.282 (0.219–0.363)
	DN AUC_0-t_ ([ng·h/mL]/mg)	1.107	1.498	—	19.6	DR / ER-MPH / IR MPH	0.739 (0.635–0.860)

^a^ Within the limits of bioequivalence (0.8–1.25).

DR/ER-MPH, delayed-release and extended-release methylphenidate; IR MPH, immediate-release methylphenidate; DN, dose-normalized; C_max_, peak observed plasma concentration; AUC_0-t_, area under the concentration–time curve from zero (predose) to time of last quantifiable concentration; LS, least squares; CV, coefficient of variation; CI, confidence interval; NR, not reported.

### Morning food effect

**FIG. 2.**
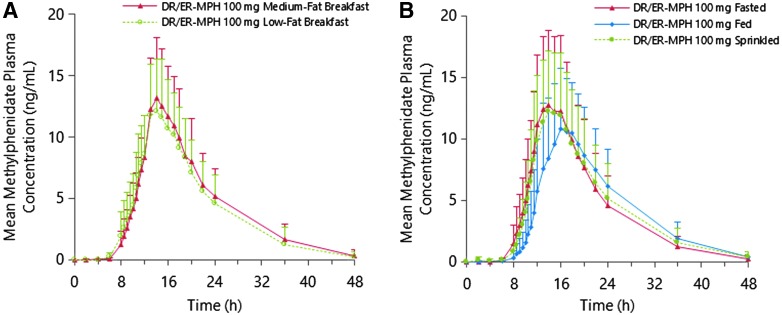
**(A)** Mean methylphenidate plasma concentrations after single evening doses of 100-mg DR/ER-MPH followed by low- and medium-fat breakfasts (*n* = 13) in Study I (Part 2); **(B)** mean methylphenidate plasma concentration after single evening doses of 100-mg DR/ER-MPH in fed, sprinkled, and fasted states followed by a high-fat breakfast (*n* = 18) in Study II. Error bars represent + standard deviation of the mean. DR/ER-MPH, delayed-release and extended-release methylphenidate.

To evaluate the effect of breakfast content on the PK profile of DR/ER-MPH, 13 participants who completed Part 1 of Study I received a subsequent evening dose of 100-mg DR/ER-MPH followed by a low-fat breakfast the next morning instead of a medium-fat breakfast. When evening-dosed DR/ER-MPH was followed by a medium-fat (Part 1) versus a low-fat (Part 2) breakfast, the shape of the absorption profiles (Fig. 2A) largely overlapped and PK parameters were similar (Table 4). The average value for AUC_0-t_ and C_max_ was 7.7% and 3.9% higher, respectively, with the medium-fat versus the low-fat breakfast. The geometric LS mean ratios (low fat vs. medium fat) for AUC_0-t_ and C_max_ were 0.923 (90% CI: 0.733–1.164) and 0.970 (90% CI: 0.773–1.217) (Table 3), respectively. The lower CI bounds for both PK parameters fell below the standard 0.8–1.25 bioequivalence range, indicating a slightly increased peak and extent of absorption of MPH with a medium-fat versus a low-fat breakfast.

**Table 4.** Methylphenidate PK Parameters After Single Evening Doses of 100-mg DR/ER-MPH Followed by a Medium- or Low-Fat Breakfast (Study I, Part 2)

	*DR/ER-MPH 100 mg*	
*Parameter*	*Medium-fat breakfast* n* = 13*	*Low-fat breakfast* n* = 13*
Mean C_max_ (ng/mL) ± CV (%)	13.56 ± 34.1	13.05 ± 31.8
Mean AUC_0-t_ (ng·h/mL) ± CV (%)	183.8 ± 31.9	170.7 ± 34.5
Mean AUC_0-∞_ (ng·h/mL) ± CV (%)	190.2 ± 33.1	173.7 ± 36.3
Mean T_max_ (h) ± CV (%)	14.31 ± 6.6	14.24 ± 10.0
Median T_max_ (h) (range)	14.00 (13.00–17.00)	14.00 (13.00–17.00)
Mean t_1/2_ (h) ± CV (%)	6.54 ± 37.9	5.35 ± 36.1
Mean λ_z_ (1/h) ± CV (%)	0.1184 ± 31.0	0.1394 ± 22.1

DR/ER-MPH, delayed-release and extended-release methylphenidate; C_max_, peak observed plasma concentration; AUC_0-t_, area under the concentration–time curve from zero (predose) to time of last quantifiable concentration; AUC_0-∞_, area under the concentration–time curve from zero (predose) extrapolated to infinite time; T_max_, time to peak observed plasma concentration; t_1/2_, terminal phase half-life; CV, coefficient of variation; PK, pharmacokinetic.

### Evening food effect

The mean plasma MPH concentration–time profiles after evening administration of a single 100-mg dose of DR/ER-MPH under fed, sprinkled, and fasted states are shown in Figure 2B. The concentration–time profiles and PK parameters (Table 5) were highly consistent between the fasted and sprinkled states. Evening dosing of 100-mg DR/ER-MPH in the fed state resulted in 11% and 14% lower C_max_ than the sprinkled and fasted states, respectively, and median T_max_ was 2.5 hours longer for the fed state versus both the sprinkled and fasted states. Mean AUC_0-t_, AUC_0-∞_, and t_1/2_ were similar among the three dosing conditions. Under all three dosing conditions, early drug exposure was minimal: MPH exposure from 0 to 10 hours was 1.3%, 3.2%, and 4.5% of total exposure for the fed, sprinkled, and fasted states, respectively.

**Table 5.** Methylphenidate PK Parameters After Single Evening Doses of 100-mg DR/ER-MPH in Fed, Sprinkled, and Fasted States (Study II)

	*DR/ER-MPH 100 mg*		
*Parameter*	*Fed* n* = 18*	*Sprinkled* n* = 18*	*Fasted* n* = 18*
Mean C_max_ (ng/mL) ± CV (%)	12.21 ± 41.3	13.71 ± 39.5	14.17 ± 46.5
Mean AUC_0-t_ (ng·h/mL) ± CV (%)	174.8 ± 41.9	182.5 ± 39.6	179.8 ± 44.7
Mean AUC_0-∞_ (ng·h/mL) ± CV (%)	178.7 ± 43.4	187.4 ± 40.0	183.0 ± 44.3
Mean T_max_ (h) ± CV (%)	16.67 ± 11.3	14.64 ± 14.2	14.31 ± 11.9
Median T_max_ (h) (range)	16.50 (13.00–20.00)	14.00 (11.50–20.02)	14.00 (11.50–18.05)
Mean t_1/2_ (h) ± CV (%)	5.94 ± 23.5	6.25 ± 27.2	5.90 ± 41.6
Mean λ_z_ (1/h) ± CV (%)	0.1216 ± 19.1	0.1187 ± 27.0	0.1307 ± 28.8

DR/ER-MPH, delayed-release and extended-release methylphenidate; C_max_, peak observed plasma concentration; AUC_0-t_, area under the concentration–time curve from zero (predose) to time of last quantifiable concentration; AUC_0-∞_, area under the concentration–time curve from zero (predose) extrapolated to infinite time; T_max_, time to peak observed plasma concentration; t_1/2_, terminal phase half-life; λ_z_, terminal phase rate constant; CV, coefficient of variation; PK, pharmacokinetic.

The bioequivalence analysis for the fed, sprinkled, and fasted states is summarized in Table 3. The geometric LS mean ratios for AUC_0-t_ were 0.972 (90% CI: 0.885–1.067), 1.027 (90% CI: 0.936–1.128), and 0.946 (90% CI: 0.861–1.039) for fed versus fasted, sprinkled versus fasted, and fed versus sprinkled comparisons, respectively, indicating bioequivalence between the three conditions for total MPH exposure. For C_max_, the lower CI bound of the geometric LS mean ratio for the fed state compared with both the fasted (0.866 [90% CI: 0.769–0.975]) and sprinkled states (0.885 [90% CI: 0.786–0.996]) fell below the limit for bioequivalence (Table 3). Together, the bioequivalence analyses indicated that a high-fat evening meal coinciding with evening DR/ER-MPH administration does not alter the extent of MPH exposure; peak exposure was reduced by ≤14%, which is not considered to be clinically meaningful. Moreover, sprinkling capsule contents had no effect on PK parameters versus administration of an intact capsule.

### Relative bioavailability

**FIG. 3.**
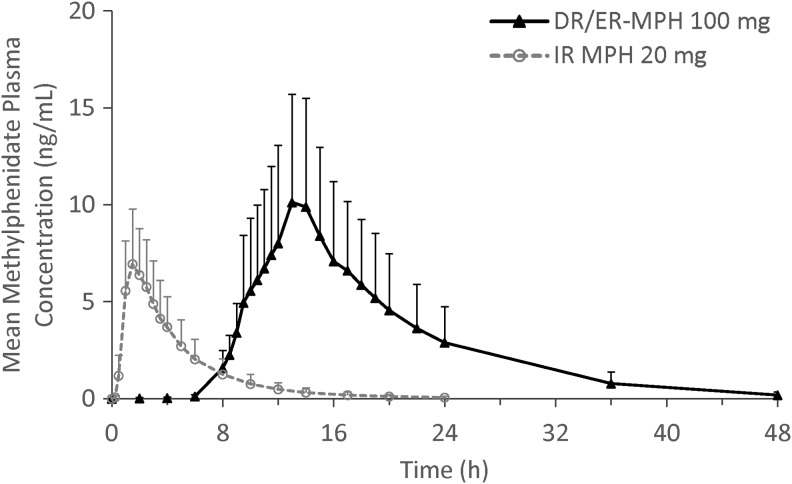
Mean methylphenidate plasma concentrations after single evening doses of 100-mg DR/ER-MPH (*n* = 11) and 20-mg IR MPH (*n* = 12) in Study III. Error bars represent + standard deviation of the mean. DR/ER-MPH, delayed-release and extended-release methylphenidate; IR MPH, immediate-release methylphenidate.

Mean MPH concentration–time profiles for a single evening 100-mg dose of DR/ER-MPH and a single morning 20-mg dose of IR MPH are shown in Figure 3, and plasma MPH PK parameters are summarized in Table 6. The mean T_max_ for DR/ER-MPH and IR MPH were 13.41 and 1.42 hours, respectively, and the mean t_1/2_ was ∼2.2 hours longer after a single dose of DR/ER-MPH relative to IR MPH. Mean C_max_ and AUC_0-t_ were ∼1.5 and 4 times higher, respectively, after 100 mg of DR/ER-MPH compared with 20 mg of IR MPH (Table 6). However, DN C_max_ for DR/ER-MPH was 28.2% that of IR MPH, and DN AUC_0-t_ for DR/ER-MPH was 73.9% that of IR MPH (Table 3). The 90% CI limits for the geometric LS mean ratios for both DN C_max_ and DN AUC_0–t_ were outside the limits of bioequivalence (0.8–1.25) (Table 3).

**Table 6.** Methylphenidate PK Parameters After a Single Evening Dose of 100-mg DR/ER-MPH and Morning Dose of 20-mg IR MPH (Study III)

*Parameter*	*DR/ER-MPH 100 mg* n* = 11*	*IR MPH 20 mg* n* = 12*
Mean C_max_ (ng/mL) ± CV (%)	10.46 ± 53.9	7.05 ± 40.3
Mean DN C_max_ ([ng/mL]/mg) ± CV (%)	0.105 ± 53.9	0.352 ± 40.3
Mean AUC_0-t_ (ng·h/mL) ± CV (%)	120.0 ± 52.8	32.3 ± 46.3
Mean DN AUC_0-t_ ([ng·h/mL]/mg) ± CV (%)	1.20 ± 52.8	1.62 ± 46.3
Mean AUC_0-∞_ (ng·h/mL) ± CV (%)	122.0 ± 52.2	32.7 ± 46.7
Mean T_max_ (h) ± CV (%)	13.41 ± 8.6	1.42 ± 25.3
Median T_max_ (h) (range)	14.00 (10.50–15.00)	1.50 (1.00–2.00)
Mean t_1/2_ (h) ± CV (%)	6.02 ± 34.9	3.79 ± 19.7
Mean λ_z_ (1/h) ± CV (%)	0.125 ± 25.8	0.191 ± 23.8

DR/ER-MPH, delayed-release and extended-release methylphenidate; IR MPH, immediate-release methylphenidate; DN, dose-normalized; C_max_, peak observed plasma concentration; AUC_0-t_, area under the concentration–time curve from zero (predose) to time of last quantifiable concentration; AUC_0-∞_, area under the concentration–time curve from zero (predose) extrapolated to infinite time; T_max_, time to peak observed plasma concentration; t_1/2_, terminal phase half-life; λ_z_, terminal phase rate constant; CV, coefficient of variation; PK, pharmacokinetic.

### Low intrasubject and intersubject variability

Intrasubject variability was assessed in a *post hoc* analysis using PK data from Study I Part 1. Because MPH exposure after 20- and 100-mg DR/ER-MPH doses was dose proportional, the DN PK parameters were used to compare variability within individuals, as measured by CV of the geometric LS means (Table 3). The PK parameters of DR/ER-MPH in adults exhibited low intrasubject variability, with CVs of 20.06% for C_max_ and 12.42% for AUC_0-t_. The PK parameters of DR/ER-MPH in adults also exhibited low intersubject variability in time to peak concentration, as demonstrated by the low CVs for mean T_max_, which ranged from 6.6% to 14.2% across studies (Tables 2 and 4–6).

### Multiple-dose simulation

**FIG. 4.**
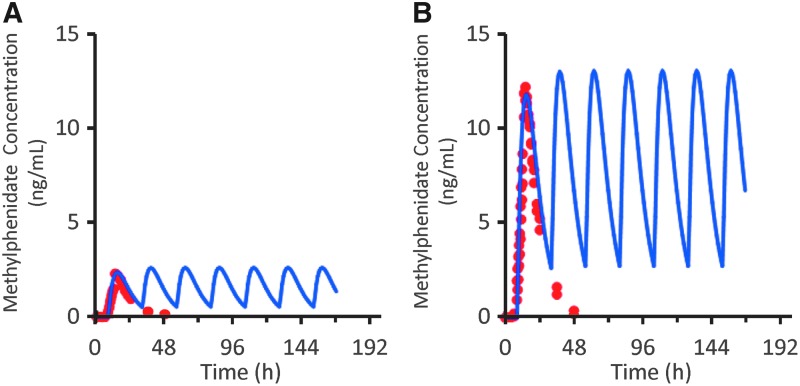
Concentration–time profile of the observed single-dose PK profile and the simulated multiple-dose PK profile of DR/ER-MPH **(A)** 20 mg and **(B)** 100 mg. Red circles show observed single-dose PK profile of DR/ER-MPH from Study I; blue lines show the simulated multiple-dose PK profile. PK, pharmacokinetic; DR/ER-MPH, delayed-release and extended-release methylphenidate.

As shown in Figure 4, a first-order, one-compartment model with a lag time adequately described the PK profile of DR/ER-MPH after oral administration, and was therefore used to predict the DR/ER-MPH PK profile after multiple once-daily dosing. The simulated multiple-dose PK profiles of 20 and 100 mg of DR/ER-MPH indicated that steady state is reached with the second dose, and there was no obvious accumulation of DR/ER-MPH. The simulated C_max_ values after a single dose and at steady state were 2.34 and 2.60 ng/mL, respectively, for 20 mg, and 11.7 and 13.0 ng/mL, respectively, for 100 mg. The accumulation ratio based on simulated C_max_ was 1.11 for both 20 and 100 mg. Similarly, simulated C_min_ values (at 24 hours postdose) after a single dose and at steady state were 1.27 and 1.34 ng/mL for 20 mg, respectively, and 6.36 and 6.69 ng/mL, respectively, for 100 mg. The accumulation ratios based on simulated C_min_ were 1.05 for 20 mg and 1.06 for 100 mg.

### Safety

All participants who received at least one dose of DR/ER-MPH were included in the safety evaluation. No serious AEs were reported. Table 7 summarizes the AEs that were reported in two or more participants in any treatment group. Vital sign changes were consistent with those expected for MPH, including increased mean pulse rates, starting ∼2–3 hours before T_max_ and lasting through 24 hours postdose. No other safety effects were noted in the laboratory, ECG, or suicidality findings.

**Table 7.** Adverse Events Reported in ≥2 Participants in Any Group

	*Study I—dose proportionality and morning food effect*			*Study II—evening food effect*			*Study III—comparative bioavailability*	
	*20 mg medium fat* n* = 20*	*100 mg medium fat* n* = 20*	*100 mg low fat* n* = 13*	*100 mg fed* n* = 24*	*100 mg sprinkled* n* = 24*	*100 mg fasted* n* = 24*	*100 mg DR/ER-MPH* n* = 11*	*20 mg IR MPH* n* = 12*
Participants with AEs^a^, *n* (%)	6 (30.0)	6 (30.0)	2 (15.4)	8 (33.3)	12 (50.0)	10 (41.7)	4 (36.4)	2 (16.7)
General disorders and administration site conditions								
Pyrexia	0	3 (15.0)	2 (15.4)	0	0	0	0	1 (8.3)
Cardiac disorders								
Palpitations	0	0	0	1 (4.2)	4 (16.7)	5 (20.8)	1 (9.1)	0
Gastrointestinal disorders								
Nausea	0	0	0	0	1 (4.2)	2 (8.3)	0	0
Metabolism and nutrition disorders								
Decreased appetite	0	2 (10.0)	0	0	2 (8.3)	4 (16.7)	0	0
Nervous system disorders								
Dizziness	0	0	0	0	2 (8.3)	2 (8.3)	2 (18.2)	0
Headache	3 (15.0)	0	0	4 (16.7)	3 (12.5)	2 (8.3)	2 (18.2)	0
Tic	0	0	0	0	2 (8.3)	1 (4.2)	0	0
Psychiatric disorders								
Hypervigilance	0	0	1 (7.7)	0	1 (4.2)	4 (16.7)	0	1 (8.3)
Skin and subcutaneous tissue disorders								
Ecchymosis	1 (5.0)	1 (5.0)	0	0	2 (8.3)	0	1 (9.1)	0

^a^ Subjects who had the same event more than once were counted only once for the preferred term.

DR/ER-MPH, delayed-release and extended-release methylphenidate; AEs, adverse events; IR MPH, immediate-release methylphenidate.

In Study I, all enrolled participants completed the study. In Study II, two participants were discontinued after the fed treatment: one participant due to a mild abnormal liver function test, which was assessed by the investigator as likely related to the study drug, and another due to a severe tooth abscess, which was assessed as unlikely related to the study drug. One participant was discontinued in the fasted group due to mild dizziness, chest discomfort, circadian rhythm sleep disorder, anorexia, moderate nausea, and headache, all assessed by the investigator as likely related to the study drug. No subjects discontinued in Study III. Together, the AE profiles and elevated pulse rates observed in these studies were consistent with the expected pharmacological results of the sympathomimetic mechanism of action of MPH.

## Discussion

In three Phase 1 studies in healthy adult volunteers, evening-dosed DR/ER-MPH exhibited a consistent and predictable delay in initial MPH release followed by a period of extended, controlled release. The PK profiles of the to-be-marketed formulation of DR/ER-MPH reported in these adult studies were highly consistent with a PK study using an earlier DR/ER-MPH formulation in healthy adults and youth with ADHD (Childress et al. 2018). These results demonstrate that the composition of the DR and ER layers, which was unchanged by the altered manufacturing process, dictates the release profile of the final formulation and not the drug core. These adult studies provided the opportunity to intensively sample around the time of initial release (i.e., twice-hourly sampling between 8 and 12 hours postdose compared with once-hourly sampling during the same period in Childress et al. 2018), allowing for greater temporal resolution to better estimate release variability. The improved temporal resolution confirmed no significant release of MPH in the first 10 hours after evening dosing of DR/ER-MPH (≤4.5%), similar to the extent of early drug exposure (<3%) noted previously in healthy adults and youth with ADHD (Childress et al. 2018). The PK exposure between the lowest (20 mg) and highest proposed doses (100 mg) was proportional to dose administered, and MPH plasma concentration–time curves were near superimposable after dose normalization, indicating that a predictable increase in systemic exposure of MPH can be expected with increasing doses of DR/ER-MPH.

For a once-daily, evening-dosed formulation, reliably delaying the initial MPH release until the early morning is hypothesized to be critical for tolerability and consistent efficacy upon awakening; furthermore, predictable release and absorption throughout the day are needed for sustained benefit. Food can alter drug exposure in several ways, including by delaying gastric emptying, stimulating bile flow, altering gastrointestinal pH, increasing splanchnic blood flow, or physically and/or chemically interacting with the drug (US Food and Drug Administration 2002). Because of its unique evening administration and DR and ER delivery, the effect of food content on DR/ER-MPH bioavailability was investigated at two time points: (1) coinciding with evening administration and (2) breakfast the following morning. Minimal morning food effect was predicted because DR/ER-MPH beads are expected to have transited to the colon. Although few food-induced physiological alterations are expected to affect bioavailability of the drug released into the colon, increased splanchnic blood flow can alter drug absorption by increasing the diffusion potential across the colonic mucosa (DeHaven and Connor 2014). In Study I, mean concentration–time curves were nearly superimposable for 100 mg doses of DR/ER-MPH regardless of meal composition (Fig. 2A), indicating that breakfast content had no impact on the absorption of MPH.

When food effect in the evening was assessed, the sprinkled and fasted states were shown to be bioequivalent in terms of both C_max_ and AUC_0-t_, as the 90% CIs for the C_max_ and AUC_0-t_ geometric LS mean ratios for the sprinkled/fasted states fell within the bioequivalence limits of 0.8–1.25 (Table 3). Thus, sprinkling DR/ER-MPH capsule contents on food gives an alternate dosing option to patients who may have difficulties swallowing intact capsules. A high-fat meal, recommended for food effect studies to maximize any potential effect (US Food and Drug Administration 2002), did not affect total exposure in terms of AUC_0-t_ compared to the sprinkled and fasted states. Moreover, mean total exposure during the first 10 hours after evening dosing was low for the three feeding groups (≤4.5%), suggesting minimal release of MPH overnight and consistent timing of morning absorption regardless of food intake. After administration with a high-fat evening meal, C_max_ was reduced by 11%–14% compared with the fasted and sprinkled states. For the majority of individuals, the reduction in C_max_ is not expected to be clinically relevant, given that the predicted therapeutic range of plasma MPH concentration is well below C_max_ (Volkow et al. 1998; Swanson et al. 1999). Compared to administration in fasted states, administration of DR/ER-MPH with a high-fat evening meal delayed T_max_ by ∼2.5 hours. Since evening administration time and dosage strength of DR/ER-MPH must be individually titrated to optimize next-day efficacy and tolerability, careful dosing titration should result in optimal efficacy from early morning until the evening despite feeding condition. During and after titration, evening DR/ER-MPH should be administered consistently with or without food.

Several formulation features of DR/ER-MPH contribute to its consistent MPH release rates despite altered evening food intake. Tiny microbeads (<1 mm in diameter), such as DR/ER-MPH, are rapidly emptied from the stomach (Davis et al. 1986) and, as such, expected to be minimally affected by food-induced effects on gastric emptying (Nagavelli et al. 2010). Based on the properties of the DR and ER layers, initial dissolution and absorption of MPH are not dependent on any single factor, such as pH or variations in gastrointestinal transit (Childress et al. 2018), both factors that can be affected by food. The lack of reliance on a single trigger for release may also contribute to the low intrasubject and intersubject variability seen with DR/ER-MPH. Low intrasubject variability was observed herein in mean C_max_ and AUC_0-t_ (CV: 20.06% and 12.42%, respectively) when DR/ER-MPH was administered in a fasted state. Moreover, low intersubject variability was observed in mean T_max_ (CV: 6.6%–14.2%) when compared between individuals in the same treatment group (i.e., under identical feeding conditions), which is consistent with the previous PK studies on DR/ER-MPH, in which low intersubject variability in T_max_ (CV ≤14.5%) and, importantly, the mean time to reach plasma MPH concentrations of 2–5 ng/mL on the ascending concentration curve were reported (CV ≤17.7%) (Childress et al. 2018). Therefore, despite the delay in T_max_ when DR/ER-MPH is administered with a high-fat meal, the low intersubject variability in initial to peak absorption between individuals in the same feeding state suggests that optimal titration of DR/ER-MPH when administered consistently with or without food will result in consistent exposure.

Simulations of multiple DR/ER-MPH dosing were performed to predict whether DR/ER-MPH would accumulate with repeated dosing. Predicted accumulation ratios were ≤1.11 at steady state, consistent with the accumulation ratio of 1.14 observed in a multiple-dose study of another ER MPH formulation, osmotic-release oral system (OROS) MPH (Modi et al. 2000a). Therefore, despite the extended elimination phase of DR/ER-MPH relative to OROS MPH and other MPH formulations (Maldonado et al. 2013; Childress et al. 2016), accumulation is predicted to be negligible with repeated dose administration; therefore, the findings of the single-dose PK studies are expected to be consistent with repeated dosing.

Compared to IR MPH, DR/ER-MPH exhibited a protracted elimination phase due to its targeted delivery to the relatively less absorptive colon (Kimura et al. 1994). Colonic delivery likely also contributes to the decreased bioavailability of MPH (73.9% based on DN AUC_0-t_) from a single dose of DR/ER-MPH relative to a single dose of IR MPH. Owing to the colonic mucosa being a less absorptive surface, a fraction of the released MPH is likely not absorbed and undergoes fecal elimination. This concept is consistent with the findings of a study evaluating the relative bioavailability of colon infusions of MPH versus oral administration, where colonic infusions resulted in a relative bioavailability of 67.8%–74.5% compared with the oral dose (ALZA Corporation, 2000).

The results of these PK studies should be considered in light of their potential limitations. First, as these studies were not designed to evaluate the clinical efficacy of DR/ER-MPH, caution should be exercised when generalizing the PK findings reported herein to next-day efficacy in patients with ADHD. Second, while the studies had small sample sizes, they are characteristic of studies investigating PK parameters. Third, these studies were performed in healthy adults; however, a previous study showed similar weight-adjusted PK properties of DR/ER-MPH between healthy adults and adolescents and children with ADHD (Childress et al. 2018). While an absence of food effect has been shown consistently with another ER MPH formulation (i.e., OROS MPH), in studies of children with ADHD (Wigal et al. 2011) and healthy adults (Modi et al., 2000b; Auiler et al., 2002), this was not studied herein. Fourth, because DR/ER-MPH was administered as a single dose under strict conditions, results may not necessarily reflect real-world variability and tolerability. Despite these limitations, DR/ER-MPH was well tolerated by healthy adults, with AEs consistent with the established safety profile of MPH, and no serious AEs or sleep-related AEs were reported. Fifth, the prediction of negligible accumulation of MPH upon repeated dosing of DR/ER-MPH is based on modeling and not a multiple-dose PK study.

The PK profile of DR/ER-MPH reported here and previously (Childress et al. 2018) is characterized by a predictable and consistent delay, resulting in early morning release of MPH that extends into the afternoon and evening. This PK profile corresponds with the recently published clinical findings of a pivotal Phase 3 trial conducted in a naturalistic setting with a 3-week, forced-dose titration design (Pliszka et al. 2017). Based on the absence of clinically significant food effects reported here, the pivotal trial did not restrict the type of food consumed. In this naturalistic setting, 3 weeks of DR/ER-MPH treatment resulted in consistent improvements in ADHD symptoms throughout the day and ADHD-related functional impairment from early morning through to the evening versus placebo despite variable dietary conditions. Consistent with negligible overnight release of MPH and predicted lack of accumulation with multiple dosing reported herein, the safety profile of DR/ER-MPH in the pivotal study reflected the known safety profile of existing MPH formulations.

## Conclusions

DR/ER-MPH demonstrated dose-proportional PKs between the 20- and 100-mg doses. The PK profile of DR/ER-MPH was not meaningfully affected by low- versus medium-fat breakfast content the morning after evening dosing or by sprinkling on food versus intact capsule administration. Due to its delivery to the relatively less absorptive colon, DR/ER-MPH displayed an extended elimination phase throughout the following day and decreased DN bioavailability compared to IR MPH. Despite the protracted elimination phase, multiple-dose simulations showed no evidence of accumulation.

## Clinical Significance

Evening-dosed DR/ER-MPH represents a shift in the approach to the timing of MPH delivery to address the unmet need for a once-daily ADHD medication that provides efficacy upon awakening and into the evening (Pliszka et al. 2017). In these PK studies, evening-dosed DR/ER-MPH had no clinically meaningful nighttime drug exposure until after 10 hours following administration. This delayed onset was consistent across 20- and 100-mg doses, and was not affected by evening or morning food intake. Larger doses of DR/ER-MPH resulted in proportionally higher plasma MPH concentration, which allows clinicians to titrate doses with relatively predictable outcome. Dosage strength and evening administration time should be adjusted and established with a regular evening routine (with or without food) to optimize tolerability and efficacy from early morning until the evening. Under identical feeding conditions, PK characteristics of DR/ER-MPH showed low intrasubject variability in T_max_, C_max_, and AUC_0-t_ and intersubject variability in T_max_, all of which should lead to consistent drug exposure with repeated dosing when taken at the prescribed time and consistently with or without meals. Negligible accumulation is predicted; therefore, the PK properties described here are not expected to be altered over time. Together, these results suggest that DR/ER-MPH provides a novel and flexible MPH formulation for the treatment of ADHD.
